# The Target Selects the Toxin: Specific Amino Acids in Snake-Prey Nicotinic Acetylcholine Receptors That Are Selectively Bound by King Cobra Venoms

**DOI:** 10.3390/toxins14080528

**Published:** 2022-08-01

**Authors:** Uthpala Chandrasekara, Richard J. Harris, Bryan G. Fry

**Affiliations:** Venom Evolution Laboratory, School of Biological Sciences, University of Queensland, St. Lucia 4072, Australia; u.chandrasekara@uq.edu.au (U.C.); rharris2727@googlemail.com (R.J.H.)

**Keywords:** venom, evolution, toxin, nicotinic acetylcholine receptor

## Abstract

Snake venom is an adaptive ecological trait that has evolved primarily as a form of prey subjugation. Thus, the selection pressure for toxin diversification is exerted by the prey’s physiological targets, with this pressure being particularly acute for specialist feeders, such as the King Cobra species, all of which are snake-prey specialists. However, while extensive research has been undertaken to elucidate key amino acids that guide toxin structure–activity relationships, reciprocal investigations into the specific sites guiding prey-lineage selective effects have been lacking. This has largely been due to the lack of assay systems amenable to systematic amino acid replacements of targeted proteins in the prey’s physiological pathways. To fill this knowledge gap, we used a recently described approach based upon mimotope peptides corresponding to the orthosteric site of nicotinic acetylcholine receptor alpha-1 subunits, a major binding site for snake venom neurotoxins that cause flaccid paralysis. We investigated the venoms of four different types of King Cobra (Cambodian, Javan, Malaysian, and Thai). This approach allowed for the determination of the key amino acid positions in King Cobra snake prey that are selectively bound by the toxins, whereby replacing these amino acids in the snake-prey orthosteric site with those from lizards or rats resulted in a significantly lower level of binding by the venoms, while conversely replacing the lizard or rat amino acids with those from the snake at that position increased the binding. By doing such, we identified three negatively charged amino acids in the snake orthosteric site that are strongly bound by the positively charged neurotoxic three-finger toxins found in King Cobra venom. This study, thus, sheds light on the selection pressures exerted by a specialist prey item for the evolution of lineage-selective toxins.

## 1. Introduction

Predator–prey interactions amongst species are often considered major evolutionary driving forces; thus, traits that intensify such interactions are believed to provide positive selection pressures [1,2]. Snake venom is considered a highly diverse ecological trait in which the selection pressures from predator–prey arms races aid in the evolution and diversification of venom systems [3,4]. The significant feature of snake venom is that it disrupts critical endophysiological processes of the victim and thus provides a benefit to the venomous animal, such as enabling prey capture [5,6,7,8,9]. Thus, it has been documented that selection upon some snake venom has allowed for prey-specific toxins to evolve, allowing for a more efficient subjugating of natural prey items [10,11,12].

This prey-specific targeting of some toxins has notably been found within three-finger toxins (3FTxs), which are ubiquitously found in venoms of the Elapidae family and also some members of the Colubridae and Lamprophiidae families [13,14,15,16,17,18,19]. These toxins predominantly target the muscle type alpha-1 nicotinic acetylcholine receptor (nAChR) [20,21], a transmembrane protein that expresses fast receptor functional activity upon binding of the small endogenous neurotransmitter acetylcholine [14]. Competitive binding of 3FTxs to the orthosteric site of nAChR would bind inhibits the binding of acetylcholine, thus disrupting the nerve–muscle signal transduction, thereby causing flaccid paralysis, leading to the death via respiratory paralysis [22,23]. The very confined binding of 3FTx at the orthosteric site of the nAChR of the targeted animal is accomplished by numerous molecular interactions among critical amino acid residues of the receptor and ligand [24,25].

Numerous studies have investigated diet-related adaptive evolutionary processes across different lineages of venomous snakes with the presence of taxon-specific crude venoms containing specific 3FTx isoforms that induce toxicity towards both natural and non-natural prey animals [10,11,26,27]. One study revealed that *Oxybelis fulgidus* (green vine snake) contains 3FTx named fulgimotoxin, which exhibits greater neurotoxic effects towards its natural prey items but no toxicity towards nonprey items [28]. Moreover, *Boiga dendrophila* (mangrove Snake), a snake that predates on birds, contains the bird-specific predatory toxin denmotoxin in its venom [29]. Iridiotoxin, which is a type of 3FTxs found in a related species, *Boiga irregularis* (brown tree snake), showed a higher toxic potency towards birds and lizards but did not show significant mammalian toxicity [30]. The Amazon puffing snake (*Spilotes sulphureus*) is lethally toxic to both mammals and lizards yet contains individual 3FTx types, sulmotoxin 1 and sulditoxin, which have differential lethality towards these prey types. Sulmotoxin 1 exhibits high lethality towards mammals, but is nontoxic to lizards, while sulditoxin exhibits the reverse pattern (high toxicity to lizards and nontoxic to mammals) [12]. Therefore, the presence of specific prey-targeting 3FTx isoforms suggests that some snake venoms are under positive selection pressures reinforced by these predator–prey arms races [31]. These selection pressures allow evolving more potent and prey-specific toxins for efficient capture of preferential prey [3,11]. Even though some studies illustrate diet-related prey-specific toxicity of snake venom through the occurrence of prey-specific 3FTx constituents, the reciprocal molecular mechanisms governing the diet-related adaptive evolution in snakes are largely undocumented. 

Since *Ophiophagus hannah* (King Cobra) venom has been shown to be highly selective for snake (*Coelognathus radiata*) alpha-1 [11], we hypothesized that the prey-specific toxicity of *O. hannah* is highly correlated with specific amino acids within the snake-prey alpha-1 orthosteric site [32]. To investigate this hypothesis, we used a well-validated novel biolayer interferometry assay [33,34] to screen the venoms of four different types of King Cobras (Cambodian, Javan, Malaysian, and Thai) using native and mutant orthosteric sites. A series of mutant mimotopes were then designed to replace specific amino acids with those of the snake, rodent, and lizard mimotopes to determine which amino acids are susceptible to that of the crude venom of *O. hannah*. The novel mimotope-based biolayer interferometry assay offers a greater advantage in understanding receptor-toxin binding affinities in a taxon-specific manner, offering invaluable opportunities to explore the interactions of these incredible toxic constituents on biological targets, such as nAChRs. 

## 2. Results and Discussion

**Figure 1:**
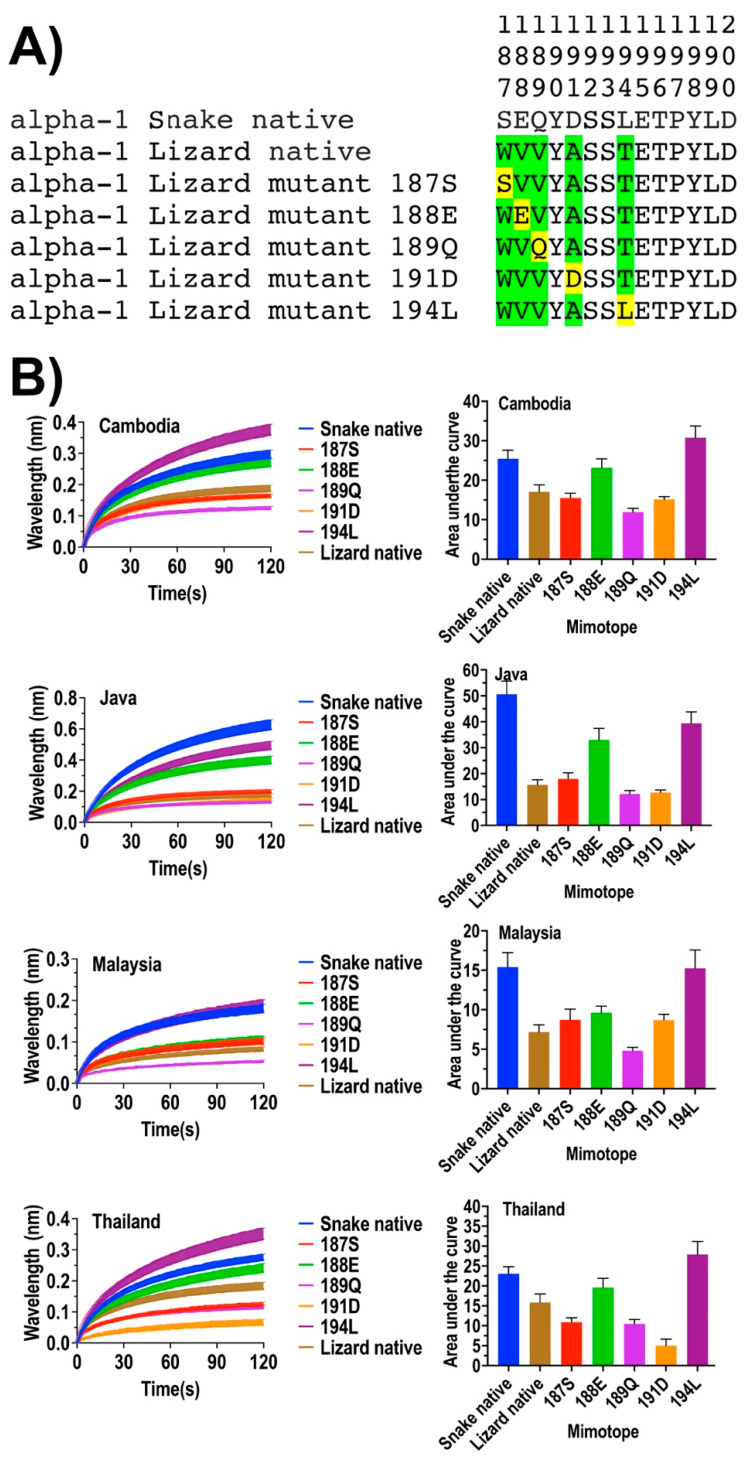
Comparison of venom binding affinities of the native and synthetic peptide mimotopes against King Cobra (*O. hannah*) venom from four geographic locales (Cambodia, Java, Malaysia, and Thailand). (**A**) Amino acid sequences at positions 187–200 of native snake and native lizard orthosteric sites, and amino acid swap mutants of the lizard orthosteric site. The amino acids highlighted in green indicate the differential amino acids of lizards with the snake. The substituted amino acids are in yellow. (**B**) Curve graphs of relative binding and bar graphs of the area under curve.

**Figure 2:**
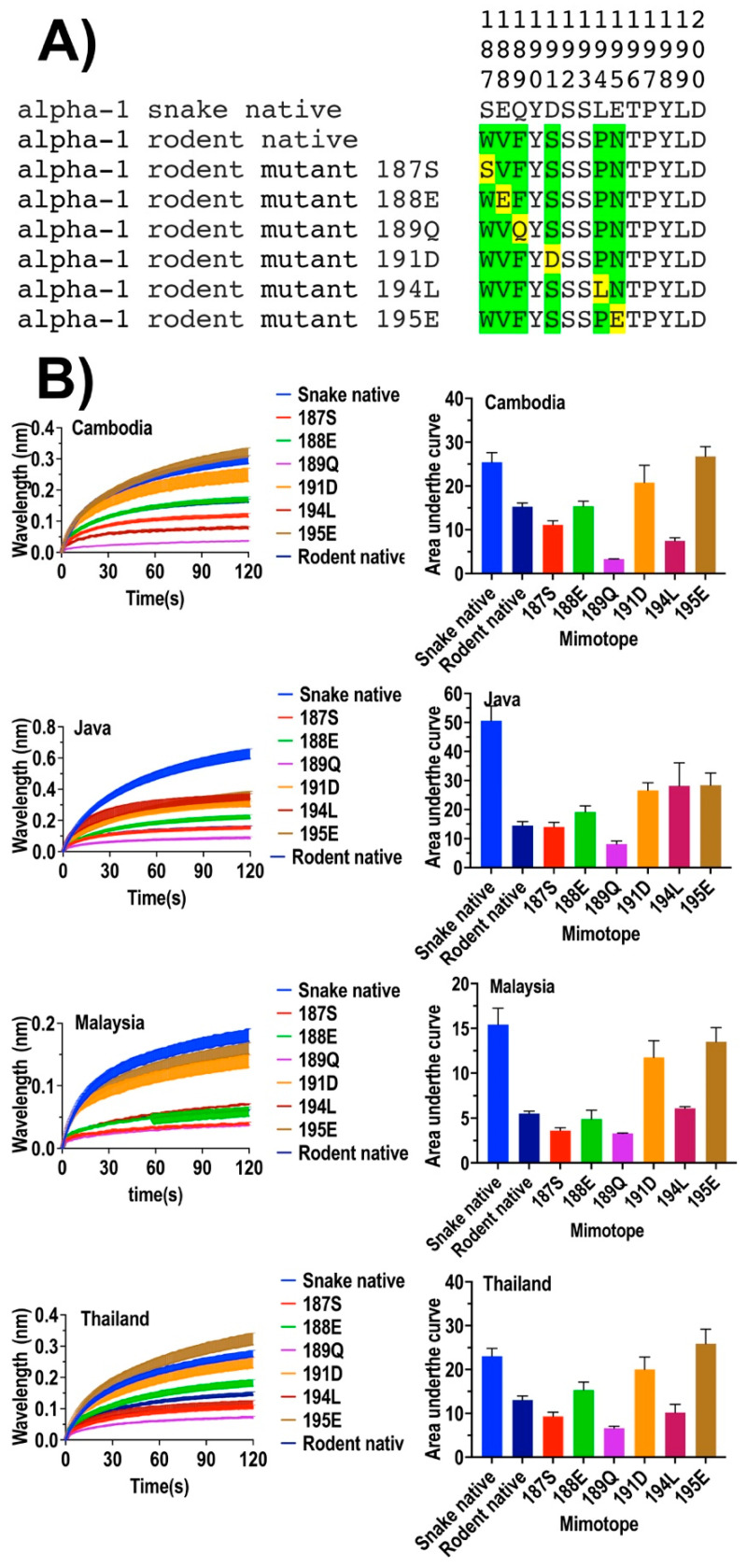
Comparison of venom binding affinities of the native and synthetic peptide mimotopes against King Cobra (*O. hannah*) venom from four geographic locales (Cambodia, Java, Malaysia, and Thailand). (**A**) Amino acid sequences at positions 187–200 of native snake and native rat orthosteric sites, and amino acid swap mutants of the rat orthosteric site. The amino acids highlighted in green indicate the differential amino acids of rat with the snake. The substituted amino acids are in yellow. (**B**) Curve graphs of relative binding and bar graphs of the area under curve.

**Figure 3:**
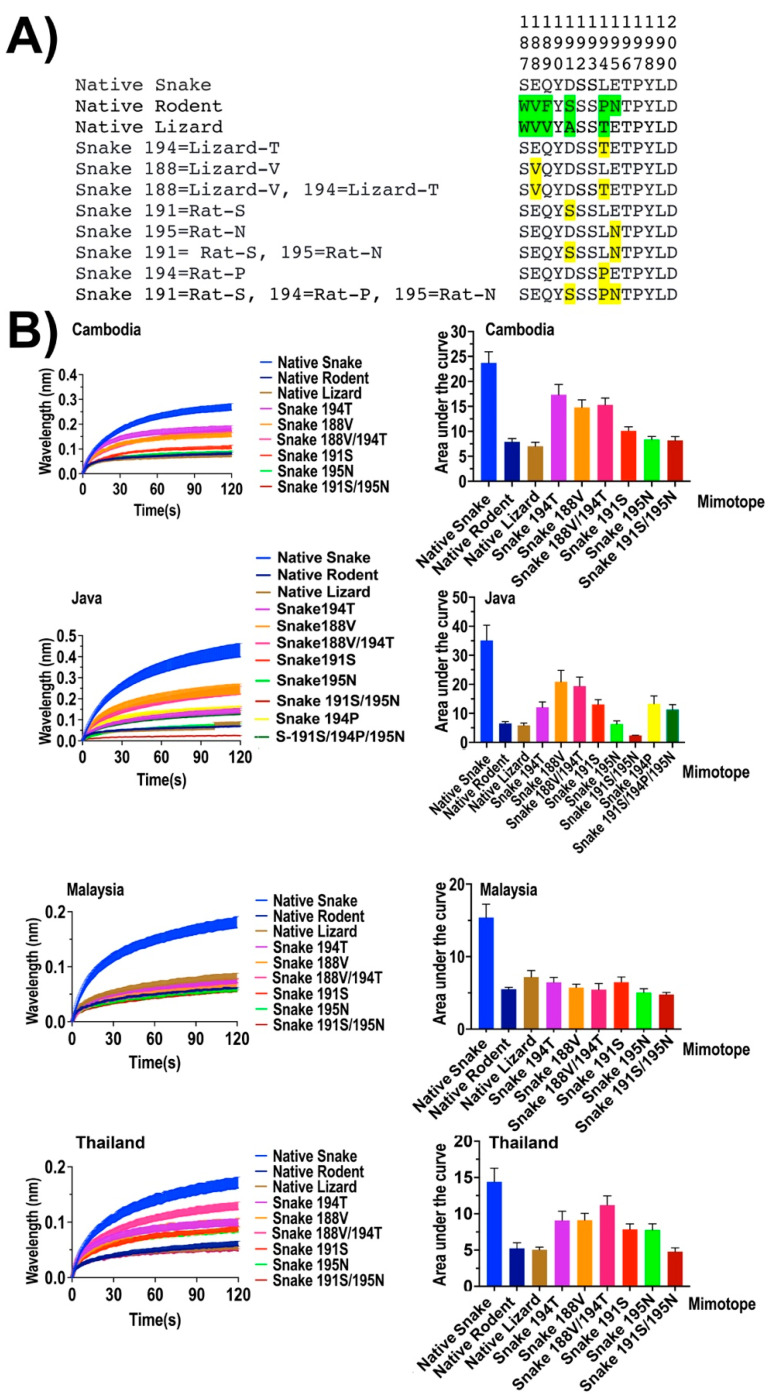
Comparison of venom binding affinities of the native and synthetic peptide mimotopes against King Cobra (*O. hannah*) venom from four geographic locales (Cambodia, Java, Malaysia, and Thailand). (**A**) Amino acid sequences at positions 187–200 of native snake, lizard, and rat orthosteric sites, and amino acid swap mutants of the snake orthosteric site. The amino acids highlighted in green indicate the differential amino acids of lizards with the snake. The substituted amino acids are in yellow. (**B**) Curve graphs of relative binding and bar graphs of the area under curve.

To identify which amino acid residues of the alpha-1 binding site are being targeted by *O. hannah* venom to produce the previously noted snake selectivity, relative to the lower binding potency against lizard and rat homologous sites [11], we constructed a series of 14-amino-acid-long native and mutated peptide mimotopes, which represent the orthosteric site (amino acid positions 187–200 of the nicotinic acetylcholine alpha-1 subunit) and assayed binding using a previously validated biolayer interferometry technique (Figure 1, Figure 2 and Figure 3) [33,34]. Mutants were constructed by replacing one by one amino acids that varied between snake and lizard or rat) at positions 187, 188, 189, 191, 194, and 195 (Figure 1 and Figure 2). Subsequently, we tested the binding affinity of native and mutant mimotopes for King Cobra venom from four geographic locales: Cambodia, Java, Malaysia, and Thailand. 

While the venoms displayed differences in relative potency against a particular mimotope, they displayed a high level of conservation of the relative binding affinity with all venoms being significantly more potent in binding native snake than native lizard (Figure 1) or native rat (Figure 2) targets. 

Amino acid replacement mutants were enlightening in regard to which residues at particular positions were preferentially bound by the King Cobra venoms. For example, the lizard mutant mimotope 194L, which had the polar, uncharged amino acid threonine (T) replaced at position 194 by the corresponding snake amino acid, nonpolar, uncharged amino acid leucine (L), showed a dramatic increase in binding by all four King Cobra venoms (Figure 1), suggestive that the polar amino acid 194L in the snake orthosteric site is a key biochemical state. However, amino acid three-dimensional chemistry is strongly influenced by steric interactions with adjacent amino acids, which was reflected by the rodent mutant mimotope 194L, which, consisting of the same single point mutation where position 194 rodent amino acid residue proline (P) was replaced with snake leucine (L), did not show any binding increase compared with the native rodent sequence except for the Javanese King Cobra venom (Figure 2). The variation between the Javan and other King Cobra venoms is suggestive of regional variation in the venoms.

Also indicative of complex interactions between adjacent amino acids was the variable response to the introduction of negatively charged amino acids at positions 188, 191, and 195. Previous works have shown that negatively charged amino acids in the orthosteric site strongly guide binding by the positively charged neurotoxins [1,2]. For the lizard, the introduction of the negatively charged amino acid glutamic acid (E) at position 188 resulted in an increase in the binding by the King Cobra venoms, but aspartic acid (D) to position 191 had no effect (the snake and lizard shared the glutamic acid (E) at position 195) (Figure 1). In contrast, for the rat, the negatively acid glutamic acid (E) at position 188 had no effect, while aspartic acid (D) at position 191 or glutamic acid (E) at position 195 both produced strong increases in binding (Figure 2). The higher relative binding to the lizard native relative to the rat native, thus, appears to be due in part to a glutamic acid (E) at position 195 in the lizard native. 

**Figure 4:**
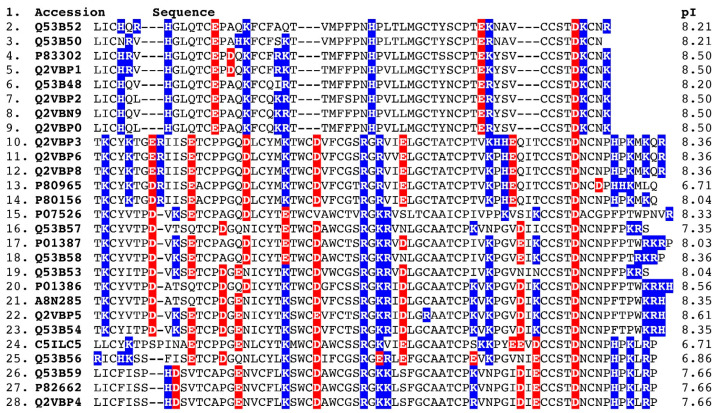
Sequence alignment and isoelectric points of King Cobra Type-I (sequences 1–9) and Type-II (sequences 10–28) alpha-neurotoxins. The amino acids highlighted in blue indicate positively charged amino acids, whereas amino acids highlighted in red indicate negatively charged amino acids.

Negatively charged amino acids in a snake-prey orthosteric site exerting a selection pressure are further evidenced by an examination of all available alpha-neurotoxic three-finger peptides available in uniprot.org (Figure 4). A total of 20 out of 27 are extremely positively charged, having isoelectric points (pI) exceeding 8, including all of the Type I (short-chain) sequences. Of the Type II (long-chain) sequences with pIs of <8, three are >7.5, and only three are <7 (6.71, 6.71, and 6.86). Thus, there is a clear trend within King Cobra venom for the presence of positively charged alpha-neurotoxins, which is congruent with the data in this study, suggesting the importance of negatively charged amino acids in the snake prey’s orthosteric site.

These results suggest that the higher toxin susceptibility in snakes is not a direct consequence of a single amino acid residue, but multiple amino acids interacting with toxins to intensify the neurotoxin susceptibility of snakes to King Cobra venom. In addition, since the interactions are quite complex, it can be postulated that these residues might act synergistically. Moreover, it can be proposed that the dynamicity of amino acid targeting between the King Cobra venoms from different locales is a consequence of different King Cobra species that inhabit different ecological niches encompassing diverse venom arsenals comprising structurally divergent 3FTx isoforms.

To test these theories further, we designed a series of reciprocal mimotopes (Figure 3). The aim was to measure whether removal of the key amino acid residues that are identified to guide the higher neurotoxic venom susceptibility of snakes could significantly lower the α-neurotoxin binding. Here, the critical amino acid residues of snakes are swapped with corresponding lizard and rodent amino acid residues one at a time to make single-mutant variants. In addition, we investigated any synergistic effect amongst these selective residues that could be intensifying the effect of neurotoxic venom susceptibility of snakes. Accordingly, a series of peptide mimotope variants of snake comprising double and triple mutations were designed to represent the possible combinations of the most selective amino acid residues localized in positions 188, 191, 194, and 195. For all four King Cobra venoms, a reduction in neurotoxin binding was observed with all snake mutant mimotopes lacking the previously identified selective residues in these positions (Figure 3).

Since the rodent mutant mimotope 194 L (position 194 of native rodent mimotope is substituted with snake leucine (L)) showed an increase in venom binding only with the King Cobra venom from the Javanese locale, we designed a snake mutant mimotope that has the rat proline at position 194 in place of leucine in the native snake and checked for the reduction of binding by the King Cobra venom from Java. The results showed a clear reduction of neurotoxin binding with the King Cobra venom from the Javanese locale. Therefore, our data further confirm that these amino acid residues in the orthosteric site of snake nAChR strongly influence the neurotoxin binding. Moreover, our data further suggest that the reduced neurotoxin susceptibility of rodents and lizards might be due to the lack of those critical amino acid residues in their orthosteric sequence.

The double-mutant snake mimotope with the rat 195S (serine) and rat 195N (asparagine) instead of the aspartic acid (D) and glutamic acid (E) at both 191 and 195 positions showed a sharp drop of neurotoxin binding compared with the point-mutated mimotopes containing single mutations at either 191 or 195 positions. This can suggest that these two negatively charged residues might act synergistically to increase toxin susceptibility. This synergistic effect is particularly strongly observed in the King Cobra venom from the Javanese locale. 

However, there was no considerable decrease in neurotoxin binding identified with the snake mutant 188 V/194 T, where both the 188 (glutamic acid (E)) and 194 (leucine (L)) critical positions of the snake orthosteric site were substituted with corresponding lizard residues of 188 valine (V) and 194 threonine (T), respectively. Therefore, it can be proposed that the two amino acids, glutamic acid (E) and leucine (L), localized in positions 188 and 194, respectively, of the snake orthosteric site might not act synergistically to strengthen the neurotoxin binding. 

Further, the triple-mutant snake mimotope 191S/194P/195N, which was constructed via swapping key amino acids of the snake at positions 191 (aspartic acid (D)), 194 (leucine (L)), and 195 (glutamic acid (E)) with corresponding rodent residues (191 serine (S), 194 proline (P), and 195 asparagine (N)), was tested to identify that all three amino acids work together to intensify the venom susceptibility of the snake to the King Cobra venom of the Javanese locale (we only tested this triple-mutant mimotope with the King Cobra venom from the Javanese locale since 194L rodent mutant showed an increase in neurotoxin binding only with the Javanese locale venom). However, the results did not show a significant decrease in binding compared with the single and double mutants, thus indicating the nonexistence of a combined effect among those three residues.

Conspicuously, our data propose that even though these critical negatively charged residues confer the increased susceptibility of snakes, the higher neurotoxin binding of snake receptors is directly or indirectly mediated by the other amino acid residues, which localize inside and outside the orthosteric site of snake nAChR; thus, the ligand–receptor molecular interactions are quite a complex process.

Consistent with previous works suggesting that prey ecology is a major influencer in shaping predatory venom composition [17], the variable neurotoxin potency of the King Cobra venom in binding the orthosteric site of snake prey versus lizard or rodent is reflective of the evolutionary adaptations, which are shaped by arms races between King Cobras and their native prey items. Our results propose that the key negatively charged amino acid residues in snake nAChR might impose a strong selection pressure on the King Cobra venom to evolve alpha-neurotoxins that are reciprocally positively charged. Further, the investigations of the orthosteric site sequences of snake, rodent, and lizard demonstrate a higher variability in variable amino acid positions. This suggests that these variable sequences are likely to impose different selection pressures on the King Cobra venom and might facilitate the evolution of specific prey-targeted 3FTxs. The venom binding data of our study demonstrate a considerably higher neurotoxic binding with native snake-prey mimotope compared with lizard and rat, which is consistent with the dietary preference of King Cobras for snakes compared with rodents and lizards.

It is important to note that though the crude King Cobra venom sourced from all four geographic localities tested in the present study demonstrated a relatively similar binding pattern (with the snake native bound the strongest), a considerable divergence in overall neurotoxic binding was exhibited among Javanese and Malaysian locals. This supports the idea of the geographical variation of venom composition and venom toxicity. Recent quantitative proteomic studies on Malaysian and Javanese King Cobra venoms showed that the crude King Cobra venom of the Javanese locale contains a comparatively higher amount of alpha-neurotoxic 3FTxs, which accounts for 64.2% of total venom proteins with ≥20 subtypes [32]. Conversely, the Malaysian King Cobra venom showed a lower percentage of 3FTxs, which makes up 43% of their total venom proteins with 13 subtypes [32]. The results obtained from our present study, thus, further reveal a piece of evidence for the compositional variation of King Cobra venom linked with geographical distribution. Similarly, increased concentrations of defensive cytotoxic L-amino acid oxidases has been noted for the Malaysian King Cobra venom; thus, the reduced neurotoxic potency of the Malaysian King Cobra venom would be due to the higher amount these defensive toxins in their venom [35]. 

In summary, we identified key negatively charged amino acid residues (188E, 191D, 195E) that guide the snake-prey preferential binding of positively charged alpha-neurotoxins in King Cobra venom. Changes made at those positions in snake receptors with corresponding rodent and lizard positions showed a drastic decrease in venom sensitivity, while lizard or rodent mutants containing these negatively charged amino acids had an increased rate of binding, confirming that these key structural residues might highly contribute to the neurotoxic sensitivity of snakes to King Cobra venom. Collectively, the findings of our study reinforce filling the gap in evolutionary biology knowledge by providing strong evidence on the diet-related adaptive evolution of snake venom systems and the selection pressure exerted by prey pathophysiological targets.

## 3. Materials and Methods

### 3.1. Venom Stock Collection and Preparation

King cobra (*Ophiophagus hannah*) venom samples (pooled) from adult captive snakes spanning four geographic locales were sourced from the long-term cryogenic collection of the Venom Evolution Lab, University of Queensland, St Lucia, Australia. All the venom study protocols of this work were performed with University of Queensland Biosafety Approval #IBC134BSBS2015 and University of Queensland Animal Ethics Approval 2021/AE000075. The lyophilized crude venom samples were reconstituted with deionized water prior to the use. The centrifugation was performed at 14,000 RCF for 10 min with a temperature of 4 °C. The pellet, if any, was discarded, and the supernatant was used to make a working venom stock of 1 mg/mL in 50% of glycerol, which was used to preserve the enzymatic action while eliminating freezing upon storage at −20 °C. The concentrations of the prepared venom stocks were checked at 280 nm with a NanoDrop 2000 UV–VIS Spectrophotometer (Thermo Fisher Scientific, Waltham, MA, USA). 

### 3.2. Mimotope Design and Preparation 

A series of 14-amino-acid-long native and mutated peptide mimotopes, which represent regions 187–200 of the alpha-1 subunit of muscle-type nAChR of snake, rodent, and lizard were designed following the previously validated protocol [33]. The point mutations were introduced by swapping each of the variable amino acid positions of rodent and lizard sequences with corresponding snake positions. Another series of snake mutant mimotopes were designed by substituting the identified key residues of the snake orthosteric site with corresponding rodent and lizard positions. Each of the mimotopes employed a biotin linker attached to two aminohexanoic acid (Ahx) spacers, forming a 30 Å linker. GenicBio Ltd. (Shanghai, China) synthesized the designed mimotopes upon specification. The amino acid sequences of alpha-1 orthosteric sites of native rodents and lizards were taken from the following accession codes: lizard alpha-1 (GenBank XM_015426640) and rodent alpha-1 (UniProt P25108). The snake alpha-1 orthosteric site amino acid sequence (*Coelognathus radiatus*) was characterized in an earlier research work and was used for the present work. The conserved cysteine residues localized at positions 192 and 193 of each of the mimotope sequences were replaced with a pair of serine molecules to eliminate the uncontrollable postsynthetic thiol oxidation. Since the highly conserved cysteine residues do not directly contribute to receptor–ligand interactions, it is anticipated that the elimination of the two tandem cysteine residues in the orthosteric sequence does not produce a considerable effect on the ligand binding. However, it has been identified that the presence of cysteines plays an important role in the structural formation of the ligand-binding site of the overall receptor. Therefore, the ligand-binding kinetics of the synthetic peptide mimotopes and the entire nicotinic acetylcholine receptor are noncomparable or should be interpreted carefully. Working mimotope stock solutions of 50 µg/mL were made by dissolving each of the supplied mimotopes of dried pellet in 100% dimethyl sulfoxide (DMSO) following 1:10 dilution with deionized water. All prepared mimotope stock solutions were stored at −20 °C for future use. 

### 3.3. Biolayer Interferometry 

The ligand–receptor binding affinities were measured utilizing a label-free, microfluidics-free novel biolayer interferometry assay, which was performed on an Octet HTX system (ForteBio, Fremont, CA, USA). All the experimental procedures of the octet potency assay along with the data acquisition and data analysis were performed according to the previously validated protocol [33]. The increased accumulation of ligand molecules at the interacting surface of the streptavidin biosensor increases the optical thickness, thus changing the light reflection pattern from the biosensor surface. This produces a quantifiable spectral shift, which will consequently result in binding kinetic information that can be quantified in real time. For the biolayer interferometry assay, the final concentration of the analyte sample was set at 50 µg/mL per well via diluting the venom stock solution in a 1:20 dilution ratio with Dulbecco’s phosphate-buffered saline (DPBS) with 0.1% BSA and 0.05% Tween-20. The stock solutions of each of the mimotopes were diluted up to the final concentration of 1 µg/mL per well with a ratio of 1:50 utilizing Dulbecco’s phosphate-buffered saline (DPBS). Before use in the biolayer interferometry assay, streptavidin biosensors were hydrated in Dulbecco’s phosphate-buffered saline (DPBS) at room temperature for 30 min with gentle rotation at 2.0 revolutions per minute (RPM). The standard acidic glycine buffer solution (10 mM glycine (pH 1.5–1.7) in ddH2O) was used as dissociation buffer to detach the bound analytes from the interacting surface of the streptavidin biosensor. Raw data are delivered with the supporting information.

### 3.4. Data Acquisition, Processing, and Statistical Analysis

All the data acquired from biolayer interferometry on a Octet HTX (ForteBio, Fremont, CA, USA) were processed in exact accordance with the previous validation protocol of the assay [33]. The raw data of the association step (performed in triplicate) were extracted from the Octet HTX system and were processed and saved as an Excel.csv file. The processed data were imported into Prism 9.2 software (GraphPad Software Inc., La Jolla, CA, USA), and calculations of the area under the curve (AUC) were made, while graphs were constructed. The ordinary one-way ANOVA with Tukey’s multiple comparisons test was performed, and all assumptions (normality of residuals and homogeneity of variance) were checked with QQ plots and Brown–Forsythe tests (please see Supplementary Files S1 and S2).

## Data Availability

All data is shown in the figures.

## Supplementary Materials

The following are available online at https://www.mdpi.com/article/10.3390/toxins14080528/s1: File S1: AUC statistics 1, File S2: AUC statistics 2.
