# Supplementary material for: The Target Selects the Toxin: Specific Amino Acids in Snake-Prey Nicotinic Acetylcholine Receptors That Are Selectively Bound by King Cobra Venoms
Source: Toxins (Basel). 2022 Aug 1;14(8):528. doi: 10.3390/toxins14080528 (PMC9416539; doi:10.3390/toxins14080528)
Supplement: Supplementary file 1 [file toxins-14-00528-s001.zip › toxins-1828338 File S1-AUC statistics 1.pdf]

toxins

MDPI

Article

The Target Selects the Toxin: Specific Amino Acids in Snake-prey Nicotinic Acetylcholine Receptors that are Selectively Bound by King Cobra Venoms

Uthpala Chandrasekara, Richard J. Harris and Bryan G Fry

Supplementary material S1 – AUC statistics 1

Test using:

Ordinary one-way ANOVA with Tukey's multiple comparisons test

All assumptions (Normality of residuals and homogeneity of variance) were checked with QQ plots and Brown-Forsythe tests.

All data met both assumptions, except the AUC bar graph of native snake, rodent and mutated mimotope with King Cobra venom from Malaysia. The QQ-plot of that indicated a small violation of normality, although an ANOVA was still used as it is robust to small violations of this normaility assumption (see the attached supplementary material 2).

AUC of native snake, native rodent and rodent mutated mimotopes with King Cobra venom from Thailand.

Tukey's multiple comparisons test	Below threshold?	Adjusted P Value
Snake native vs. 187S	Yes	<0.0001
Snake native vs. 188E	Yes	0.0036
Snake native vs. 189Q	Yes	<0.0001
Snake native vs. 191D	No	0.5765
Snake native vs. 194L	Yes	<0.0001
Snake native vs. 195E	No	0.6530
Snake native vs. Rodent native	Yes	0.0003
187S vs. 188E	Yes	0.0288
187S vs. 189Q	No	0.7102
187S vs. 191D	Yes	0.0001
187S vs. 194L	No	0.9989
187S vs. 195E	Yes	<0.0001
187S vs. Rodent native	No	0.3288
188E vs. 189Q	Yes	0.0012
188E vs. 191D	No	0.1291
188E vs. 194L	No	0.0827
188E vs. 195E	Yes	0.0001
188E vs. Rodent native	No	0.8410
189Q vs. 191D	Yes	<0.0001
189Q vs. 194L	No	0.3876
189Q vs. 195E	Yes	<0.0001
189Q vs. Rodent native	Yes	0.0177
191D vs. 194L	Yes	0.0003
191D vs. 195E	Yes	0.0353
191D vs. Rodent native	Yes	0.0091
194L vs. 195E	Yes	<0.0001

194L vs. Rodent native	No	0.6399
195E vs. Rodent native	Yes	<0.0001

AUC of native snake, native rodent and rodent mutated mimotopes with King Cobra venom from Malaysia.

Tukey's multiple comparisons test	Below threshold?	Adjusted P Value
Snake native vs. 187S	Yes	<0.0001
Snake native vs. 188E	Yes	<0.0001
Snake native vs. 189Q	Yes	<0.0001
Snake native vs. 191D	Yes	0.0204
Snake native vs. 194L	Yes	<0.0001
Snake native vs. 195E	No	0.4830
Snake native vs. Rodent native	Yes	<0.0001
187S vs. 188E	No	0.8509
187S vs. 189Q	No	>0.9999
187S vs. 191D	Yes	<0.0001
187S vs. 194L	No	0.2015
187S vs. 195E	Yes	<0.0001
187S vs. Rodent native	No	0.4816
188E vs. 189Q	No	0.6763
188E vs. 191D	Yes	<0.0001
188E vs. 194L	No	0.8923
188E vs. 195E	Yes	<0.0001
188E vs. Rodent native	No	0.9969
189Q vs. 191D	Yes	<0.0001
189Q vs. 194L	No	0.1151
189Q vs. 195E	Yes	<0.0001
189Q vs. Rodent native	No	0.3111
191D vs. 194L	Yes	0.0004
191D vs. 195E	No	0.5794
191D vs. Rodent native	Yes	0.0001
194L vs. 195E	Yes	<0.0001
194L vs. Rodent native	No	0.9980
195E vs. Rodent native	Yes	<0.0001

AUC of native snake, native rodent and rodent mutated mimotopes with King Cobra venom from Java.

Tukey's multiple comparisons test	Below threshold?	Adjusted P Value
Snake native vs. 187S	Yes	<0.0001
Snake native vs. 188E	Yes	<0.0001
Snake native vs. 189Q	Yes	<0.0001
Snake native vs. 191D	Yes	<0.0001
Snake native vs. 194L	Yes	<0.0001
Snake native vs. 195E	Yes	<0.0001
Snake native vs. Rodent native	Yes	<0.0001
187S vs. 188E	No	0.7422
187S vs. 189Q	No	0.6073
187S vs. 191D	Yes	0.0208
187S vs. 194L	Yes	0.0080
187S vs. 195E	Yes	0.0070
187S vs. Rodent native	No	>0.9999
188E vs. 189Q	No	0.0522
188E vs. 191D	No	0.3411
188E vs. 194L	No	0.1602
188E vs. 195E	No	0.1429
188E vs. Rodent native	No	0.8153
189Q vs. 191D	Yes	0.0006
189Q vs. 194L	Yes	0.0002
189Q vs. 195E	Yes	0.0002
189Q vs. Rodent native	No	0.5244
191D vs. 194L	No	0.9996
191D vs. 195E	No	0.9990
191D vs. Rodent native	Yes	0.0272
194L vs. 195E	No	>0.9999
194L vs. Rodent native	Yes	0.0105
195E vs. Rodent native	Yes	0.0092

AUC of native snake, native rodent and rodent mutated mimotopes with King Cobra venom from Cambodia.

Tukey's multiple comparisons test	Below threshold?	Adjusted P Value
Snake native vs. 187S	Yes	<0.0001
Snake native vs. 188E	Yes	0.0001
Snake native vs. 189Q	Yes	<0.0001
Snake native vs. 191D	No	0.1096
Snake native vs. 194L	Yes	<0.0001
Snake native vs. 195E	No	0.9878
Snake native vs. Rodent native	Yes	0.0001
187S vs. 188E	No	0.1750
187S vs. 189Q	Yes	0.0022
187S vs. 191D	Yes	0.0002
187S vs. 194L	No	0.3245
187S vs. 195E	Yes	<0.0001
187S vs. Rodent native	No	0.1965
188E vs. 189Q	Yes	<0.0001
188E vs. 191D	Yes	0.0466
188E vs. 194L	Yes	0.0021
188E vs. 195E	Yes	<0.0001
188E vs. Rodent native	No	>0.9999
189Q vs. 191D	Yes	<0.0001
189Q vs. 194L	No	0.1866
189Q vs. 195E	Yes	<0.0001
189Q vs. Rodent native	Yes	<0.0001
191D vs. 194L	Yes	<0.0001
191D vs. 195E	Yes	0.0230
191D vs. Rodent native	Yes	0.0408
194L vs. 195E	Yes	<0.0001
194L vs. Rodent native	Yes	0.0024
195E vs. Rodent native	Yes	<0.0001

AUC of native snake, native lizard and lizard mutated mimotopes with King Cobra venom from Thailand.

Tukey's multiple comparisons test	Below threshold?	Adjusted P Value
Snake native vs. 187S	Yes	<0.0001
Snake native vs. 188E	No	0.4044
Snake native vs. 189Q	Yes	<0.0001
Snake native vs. 191D	Yes	<0.0001
Snake native vs. 194L	No	0.1128
Snake native vs. Lizard native	Yes	0.0090
187S vs. 188E	Yes	0.0017
187S vs. 189Q	No	>0.9999
187S vs. 191D	Yes	0.0390
187S vs. 194L	Yes	<0.0001
187S vs. Lizard native	No	0.1001
188E vs. 189Q	Yes	0.0011
188E vs. 191D	Yes	<0.0001
188E vs. 194L	Yes	0.0026
188E vs. Lizard native	No	0.3241
189Q vs. 191D	No	0.0613
189Q vs. 194L	Yes	<0.0001
189Q vs. Lizard native	No	0.0645
191D vs. 194L	Yes	<0.0001
191D vs. Lizard native	Yes	0.0002
194L vs. Lizard native	Yes	<0.0001

AUC of native snake, native lizard and lizard mutated mimotopes with King Cobra venom from Malaysia.

Tukey's multiple comparisons test	Below threshold?	Adjusted P Value
Snake native vs. 187S	Yes	0.0005
Snake native vs. 188E	Yes	0.0018
Snake native vs. 189Q	Yes	<0.0001
Snake native vs. 191D	Yes	0.0004
Snake native vs. 194L	No	>0.9999
Snake native vs. Lizard native	Yes	<0.0001
187S vs. 188E	No	0.9798
187S vs. 189Q	Yes	0.0395
187S vs. 191D	No	>0.9999
187S vs. 194L	Yes	0.0006
187S vs. Lizard native	No	0.7950
188E vs. 189Q	Yes	0.0091
188E vs. 191D	No	0.9735
188E vs. 194L	Yes	0.0024
188E vs. Lizard native	No	0.3500
189Q vs. 191D	Yes	0.0430
189Q vs. 194L	Yes	<0.0001
189Q vs. Lizard native	No	0.3779
191D vs. 194L	Yes	0.0005
191D vs. Lizard native	No	0.8179
194L vs. Lizard native	Yes	<0.0001

AUC of native snake, native lizard and lizard mutated mimotopes with King Cobra venom from Java.

Tukey's multiple comparisons test	Below threshold?	Adjusted P Value
Snake native vs. 187S	Yes	<0.0001
Snake native vs. 188E	Yes	0.0002
Snake native vs. 189Q	Yes	<0.0001
Snake native vs. 191D	Yes	<0.0001
Snake native vs. 194L	Yes	0.0144
Snake native vs. Lizard native	Yes	<0.0001
187S vs. 188E	Yes	0.0012
187S vs. 189Q	No	0.3774
187S vs. 191D	No	0.4870
187S vs. 194L	Yes	<0.0001
187S vs. Lizard native	No	0.9727
188E vs. 189Q	Yes	<0.0001
188E vs. 191D	Yes	<0.0001
188E vs. 194L	No	0.2799
188E vs. Lizard native	Yes	0.0003
189Q vs. 191D	No	>0.9999
189Q vs. 194L	Yes	<0.0001
189Q vs. Lizard native	No	0.8467
191D vs. 194L	Yes	<0.0001
191D vs. Lizard native	No	0.9242
194L vs. Lizard native	Yes	<0.0001

AUC of native snake, native lizard and lizard mutated mimotopes with King Cobra venom from Cambodia.

Tukey's multiple comparisons test	Below threshold?	Adjusted P Value
Snake native vs. 187S	Yes	0.0002
Snake native vs. 188E	No	0.7422
Snake native vs. 189Q	Yes	<0.0001
Snake native vs. 191D	Yes	0.0002
Snake native vs. 194L	Yes	0.0458
Snake native vs. Lizard native	Yes	0.0013
187S vs. 188E	Yes	0.0029
187S vs. 189Q	No	0.2967
187S vs. 191D	No	>0.9999
187S vs. 194L	Yes	<0.0001
187S vs. Lizard native	No	0.9399
188E vs. 189Q	Yes	<0.0001
188E vs. 191D	Yes	0.0019
188E vs. 194L	Yes	0.0030
188E vs. Lizard native	Yes	0.0185
189Q vs. 191D	No	0.4063
189Q vs. 194L	Yes	<0.0001
189Q vs. Lizard native	No	0.0560
191D vs. 194L	Yes	<0.0001
191D vs. Lizard native	No	0.8594
194L vs. Lizard native	Yes	<0.0001

AUC of native snake, native lizard, native rodent and reciprocal mutated mimotopes with King Cobra venom from Thailand.

Tukey's multiple comparisons test	Below threshold?	Adjusted P Value
Native snake vs. Native Rodent	Yes	<0.0001
Native snake vs. Native Lizard	Yes	<0.0001
Native snake vs. S-194-L-T	Yes	0.0002
Native snake vs. S-188-L-V	Yes	0.0002
Native snake vs. S-194/188-L-T-V	Yes	0.0295
Native snake vs. S-191-R-S	Yes	<0.0001
Native snake vs. S-195-R-N	Yes	<0.0001
Native snake vs. S-191/195-R-S-N	Yes	<0.0001
Native Rodent vs. Native Lizard	No	>0.9999
Native Rodent vs. S-194-L-T	Yes	0.0061
Native Rodent vs. S-188-L-V	Yes	0.0056
Native Rodent vs. S-194/188-L-T-V	Yes	<0.0001
Native Rodent vs. S-191-R-S	No	0.1097
Native Rodent vs. S-195-R-N	No	0.1282
Native Rodent vs. S-191/195-R-S-N	No	0.9998
Native Lizard vs. S-194-L-T	Yes	0.0039
Native Lizard vs. S-188-L-V	Yes	0.0036
Native Lizard vs. S-194/188-L-T-V	Yes	<0.0001
Native Lizard vs. S-191-R-S	No	0.0737
Native Lizard vs. S-195-R-N	No	0.0867
Native Lizard vs. S-191/195-R-S-N	No	>0.9999
S-194-L-T vs. S-188-L-V	No	>0.9999
S-194-L-T vs. S-194/188-L-T-V	No	0.3046
S-194-L-T vs. S-191-R-S	No	0.8623
S-194-L-T vs. S-195-R-N	No	0.8232
S-194-L-T vs. S-191/195-R-S-N	Yes	0.0021
S-188-L-V vs. S-194/188-L-T-V	No	0.3235
S-188-L-V vs. S-191-R-S	No	0.8443
S-188-L-V vs. S-195-R-N	No	0.8030
S-188-L-V vs. S-191/195-R-S-N	Yes	0.0019
S-194/188-L-T-V vs. S-191-R-S	Yes	0.0214
S-194/188-L-T-V vs. S-195-R-N	Yes	0.0179
S-194/188-L-T-V vs. S-191/195-R-S-N	Yes	<0.0001
S-191-R-S vs. S-195-R-N	No	>0.9999
S-191-R-S vs. S-191/195-R-S-N	Yes	0.0408
S-195-R-N vs. S-191/195-R-S-N	Yes	0.0484

AUC of native snake, native lizard, native rodent and reciprocal mutated mimotopes with King Cobra venom from Malaysia.

Tukey's multiple comparisons test	Below threshold?	Adjusted P Value
Native Snake vs. Native Rodent	Yes	<0.0001
Native Snake vs. Native Lizard	Yes	<0.0001
Native Snake vs. S-194-L-T	Yes	<0.0001
Native Snake vs. S-188-L-V	Yes	<0.0001
Native Snake vs. S-194/188-L-T-V	Yes	<0.0001
Native Snake vs. S-191-R-S	Yes	<0.0001
Native Snake vs. S-195-R-N	Yes	<0.0001
Native Snake vs. S-191/195-R-S-N	Yes	<0.0001
Native Rodent vs. Native Lizard	No	0.3379
Native Rodent vs. S-194-L-T	No	0.8962
Native Rodent vs. S-188-L-V	No	>0.9999
Native Rodent vs. S-194/188-L-T-V	No	>0.9999
Native Rodent vs. S-191-R-S	No	0.8822
Native Rodent vs. S-195-R-N	No	0.9982
Native Rodent vs. S-191/195-R-S-N	No	0.9702
Native Lizard vs. S-194-L-T	No	0.9763
Native Lizard vs. S-188-L-V	No	0.5050
Native Lizard vs. S-194/188-L-T-V	No	0.2923
Native Lizard vs. S-191-R-S	No	0.9810
Native Lizard vs. S-195-R-N	No	0.1067
Native Lizard vs. S-191/195-R-S-N	No	0.0520
S-194-L-T vs. S-188-L-V	No	0.9735
S-194-L-T vs. S-194/188-L-T-V	No	0.8569
S-194-L-T vs. S-191-R-S	No	>0.9999
S-194-L-T vs. S-195-R-N	No	0.5221
S-194-L-T vs. S-191/195-R-S-N	No	0.3180
S-188-L-V vs. S-194/188-L-T-V	No	>0.9999
S-188-L-V vs. S-191-R-S	No	0.9676
S-188-L-V vs. S-195-R-N	No	0.9800
S-188-L-V vs. S-191/195-R-S-N	No	0.8881
S-194/188-L-T-V vs. S-191-R-S	No	0.8403
S-194/188-L-T-V vs. S-195-R-N	No	0.9994
S-194/188-L-T-V vs. S-191/195-R-S-N	No	0.9831
S-191-R-S vs. S-195-R-N	No	0.5002
S-191-R-S vs. S-191/195-R-S-N	No	0.3011
S-195-R-N vs. S-191/195-R-S-N	No	>0.9999

AUC of native snake, native lizard, native rodent and reciprocal mutated mimotopes with King Cobra venom from Java.

Tukey's multiple comparisons test	Below threshold?	Adjusted P Value
Native Snake vs. Native Rodent	Yes	<0.0001
Native Snake vs. Native Lizard	Yes	<0.0001
Native Snake vs. S-194-L-T	Yes	<0.0001
Native Snake vs. S-188-L-V	Yes	<0.0001
Native Snake vs. S-194/188-L-T-V	Yes	<0.0001
Native Snake vs. S-194-R-P	Yes	<0.0001
Native Snake vs. S-191-R-S	Yes	<0.0001
Native Snake vs. S-195-R-N	Yes	<0.0001
Native Snake vs. S-191/195-R-S-N	Yes	<0.0001
Native Snake vs. S-191/195/194-R-S-N-P	Yes	<0.0001
Native Rodent vs. Native Lizard	No	>0.9999
Native Rodent vs. S-194-L-T	No	0.2655
Native Rodent vs. S-188-L-V	Yes	<0.0001
Native Rodent vs. S-194/188-L-T-V	Yes	0.0001
Native Rodent vs. S-194-R-P	No	0.0929
Native Rodent vs. S-191-R-S	No	0.1136
Native Rodent vs. S-195-R-N	No	>0.9999
Native Rodent vs. S-191/195-R-S-N	No	0.6387
Native Rodent vs. S-191/195/194-R-S-N-P	No	0.4477
Native Lizard vs. S-194-L-T	No	0.1527
Native Lizard vs. S-188-L-V	Yes	<0.0001
Native Lizard vs. S-194/188-L-T-V	Yes	<0.0001
Native Lizard vs. S-194-R-P	Yes	0.0484
Native Lizard vs. S-191-R-S	No	0.0600
Native Lizard vs. S-195-R-N	No	>0.9999
Native Lizard vs. S-191/195-R-S-N	No	0.8205
Native Lizard vs. S-191/195/194-R-S-N-P	No	0.2818
S-194-L-T vs. S-188-L-V	Yes	0.0115
S-194-L-T vs. S-194/188-L-T-V	No	0.0561
S-194-L-T vs. S-194-R-P	No	>0.9999
S-194-L-T vs. S-191-R-S	No	>0.9999
S-194-L-T vs. S-195-R-N	No	0.2269
S-194-L-T vs. S-191/195-R-S-N	Yes	0.0040
S-194-L-T vs. S-191/195/194-R-S-N-P	No	>0.9999
S-188-L-V vs. S-194/188-L-T-V	No	0.9995
S-188-L-V vs. S-194-R-P	Yes	0.0411
S-188-L-V vs. S-191-R-S	Yes	0.0329
S-188-L-V vs. S-195-R-N	Yes	<0.0001
S-188-L-V vs. S-191/195-R-S-N	Yes	<0.0001
S-188-L-V vs. S-191/195/194-R-S-N-P	Yes	0.0051
S-194/188-L-T-V vs. S-194-R-P	No	0.1735
S-194/188-L-T-V vs. S-191-R-S	No	0.1438
S-194/188-L-T-V vs. S-195-R-N	Yes	0.0001
S-194/188-L-T-V vs. S-191/195-R-S-N	Yes	<0.0001
S-194/188-L-T-V vs. S-191/195/194-R-S-N-P	Yes	0.0262
S-194-R-P vs. S-191-R-S	No	>0.9999
S-194-R-P vs. S-195-R-N	No	0.0769
S-194-R-P vs. S-191/195-R-S-N	Yes	0.0011
S-194-R-P vs. S-191/195/194-R-S-N-P	No	0.9960
S-191-R-S vs. S-195-R-N	No	0.0944
S-191-R-S vs. S-191/195-R-S-N	Yes	0.0013

S-191-R-S vs. S-191/195/194-R-S-N-P	No	0.9984
S-195-R-N vs. S-191/195-R-S-N	No	0.6966
S-195-R-N vs. S-191/195/194-R-S-N-P	No	0.3939
S-191/195-R-S-N vs. S-191/195/194-R-S-N-P	Yes	0.0091

AUC of native snake, native lizard, native rodent and reciprocal mutated mimotopes with King Cobra venom from Cambodia.

Tukey's multiple comparisons test	Below threshold?	Adjusted P Value
Native Snake vs. Native Rodent	Yes	<0.0001
Native Snake vs. Native Lizard	Yes	<0.0001
Native Snake vs. S-194-L-T	Yes	0.0004
Native Snake vs. S-188-L-V	Yes	<0.0001
Native Snake vs. S-194/188-L-T-V	Yes	<0.0001
Native Snake vs. S-191-R-S	Yes	<0.0001
Native Snake vs. S-195-R-N	Yes	<0.0001
Native Snake vs. S-191/195-R-S-N	Yes	<0.0001
Native Rodent vs. Native Lizard	No	0.9955
Native Rodent vs. S-194-L-T	Yes	<0.0001
Native Rodent vs. S-188-L-V	Yes	0.0001
Native Rodent vs. S-194/188-L-T-V	Yes	<0.0001
Native Rodent vs. S-191-R-S	No	0.5257
Native Rodent vs. S-195-R-N	No	>0.9999
Native Rodent vs. S-191/195-R-S-N	No	>0.9999
Native Lizard vs. S-194-L-T	Yes	<0.0001
Native Lizard vs. S-188-L-V	Yes	<0.0001
Native Lizard vs. S-194/188-L-T-V	Yes	<0.0001
Native Lizard vs. S-191-R-S	No	0.1655
Native Lizard vs. S-195-R-N	No	0.9329
Native Lizard vs. S-191/195-R-S-N	No	0.9705
S-194-L-T vs. S-188-L-V	No	0.3683
S-194-L-T vs. S-194/188-L-T-V	No	0.6459
S-194-L-T vs. S-191-R-S	Yes	<0.0001
S-194-L-T vs. S-195-R-N	Yes	<0.0001
S-194-L-T vs. S-191/195-R-S-N	Yes	<0.0001
S-188-L-V vs. S-194/188-L-T-V	No	0.9999
S-188-L-V vs. S-191-R-S	Yes	0.0095
S-188-L-V vs. S-195-R-N	Yes	0.0004
S-188-L-V vs. S-191/195-R-S-N	Yes	0.0003
S-194/188-L-T-V vs. S-191-R-S	Yes	0.0035
S-194/188-L-T-V vs. S-195-R-N	Yes	0.0001
S-194/188-L-T-V vs. S-191/195-R-S-N	Yes	<0.0001
S-191-R-S vs. S-195-R-N	No	0.7908
S-191-R-S vs. S-191/195-R-S-N	No	0.6942
S-195-R-N vs. S-191/195-R-S-N	No	>0.9999
