# Supplementary material for: The Target Selects the Toxin: Specific Amino Acids in Snake-Prey Nicotinic Acetylcholine Receptors That Are Selectively Bound by King Cobra Venoms
Source: Toxins (Basel). 2022 Aug 1;14(8):528. doi: 10.3390/toxins14080528 (PMC9416539; doi:10.3390/toxins14080528)
Supplement: Supplementary file 1 [file toxins-14-00528-s001.zip › toxins-1828338 file S2-AUC statistics 2.pdf]

toxins

MDPI

Article

The Target Selects the Toxin: Specific Amino Acids in Snake-prey Nicotinic Acetylcholine Receptors that are Selectively Bound by King Cobra Venoms

Uthpala Chandrasekara, Richard J. Harris and Bryan G Fry

Supplementary material S2 – AUC statistics 2

- QQ plots and Brown-Forsythe tests

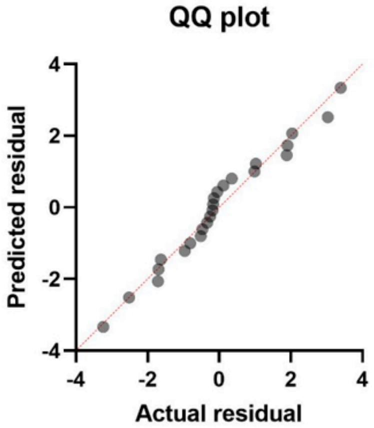

Brown-Forsythe test.	
F (DFn, DFd)	0.8819 (7, 16)
P value	0.5420
P value summary	ns
Are SDs significantly different (P < 0.05)?	No

AUC of native snake, native rodent and rodent mutated mimotopes with King Cobra venom from Thailand.

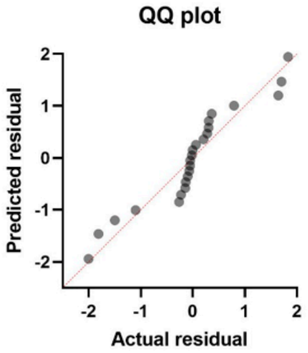

Brown-Forsythe test	
F (DFn, DFd)	1.627 (7, 16)
P value	0.1984
P value summary	ns
Are SDs significantly different (P < 0.05)?	No

AUC of native snake, native rodent and rodent mutated mimotopes with King Cobra venom from Malaysia.

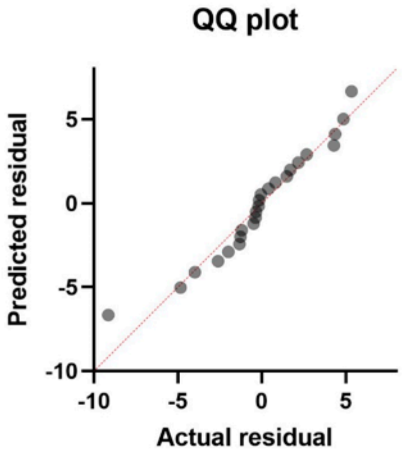

Brown-Forsythe test	
F (DFn, DFd)	0.6033 (7, 16)
P value	0.7449
P value summary	ns
Are SDs significantly different (P < 0.05)?	No

AUC of native snake, native rodent and rodent mutated mimotopes with King Cobra venom from Java.

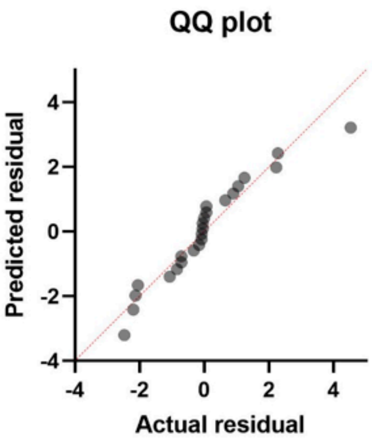

Brown-Forsythe test	
F (DFn, DFd)	0.7206 (7, 16)
P value	0.6568
P value summary	ns
Are SDs significantly different (P < 0.05)?	No

AUC of native snake, native rodent and rodent mutated mimotopes with King Cobra venom from Cambodia.

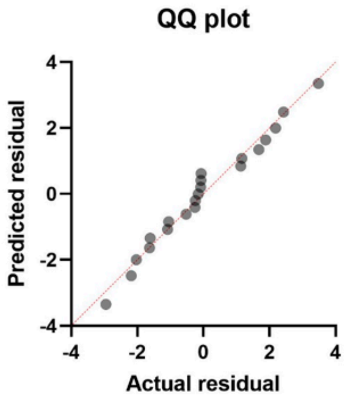

Brown-Forsythe test	
F (DFn, DFd)	0.4905 (6, 14)
P value	0.8050
P value summary	ns
Are SDs significantly different (P < 0.05)?	No

AUC of native snake, native lizard and lizard mutated mimotopes with King Cobra venom from Thailand.

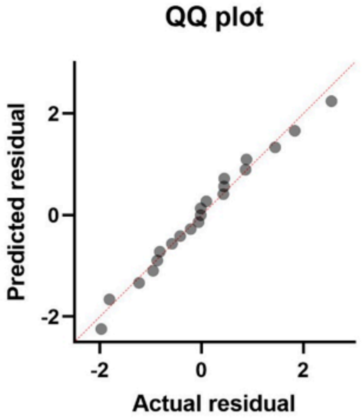

Brown-Forsythe test	
F (DFn, DFd)	0.7504 (6, 14)
P value	0.6194
P value summary	ns
Are SDs significantly different (P < 0.05)?	No

AUC of native snake, native lizard and lizard mutated mimotopes with King Cobra venom from Malaysia.

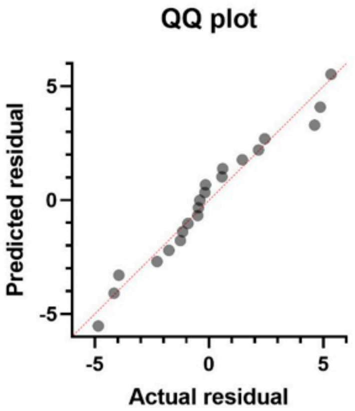

Brown-Forsythe test	
F (DFn, DFd)	0.8864 (6, 14)
P value	0.5300
P value summary	ns
Are SDs significantly different (P < 0.05)?	No

AUC of native snake, native lizard and lizard mutated mimotopes with King Cobra venom from Java.

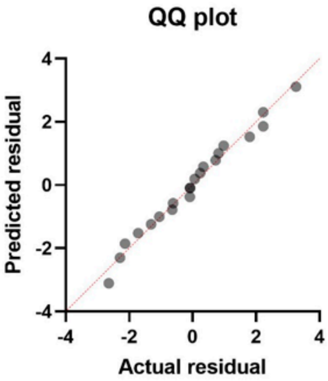

Brown-Forsythe test	
F (DFn, DFd)	0.6681 (6, 14)
P value	0.6771
P value summary	ns
Are SDs significantly different (P < 0.05)?	No

AUC of native snake, native lizard and lizard mutated mimotopes with King Cobra venom from Cambodia.

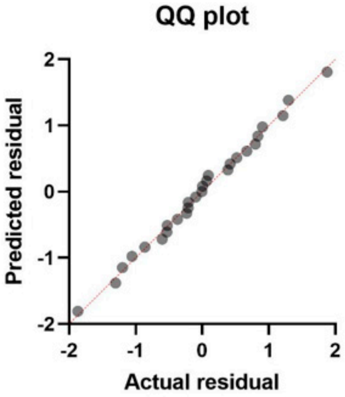

Brown-Forsythe test	
F (DFn, DFd)	0.6408 (8, 18)
P value	0.7342
P value summary	ns
Are SDs significantly different (P < 0.05)?	No

AUC of native snake, native lizard, native rodent and reciprocal mutated mimotopes with King Cobra venom from Thailand.

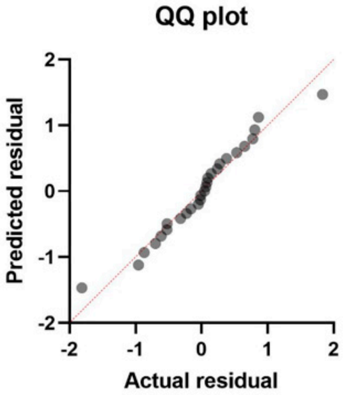

Brown-Forsythe test	
F (DFn, DFd)	1.168 (8, 18)
P value	0.3695
P value summary	ns
Are SDs significantly different (P < 0.05)?	No

AUC of native snake, native lizard, native rodent and reciprocal mutated mimotopes with King Cobra venom from Malaysia.

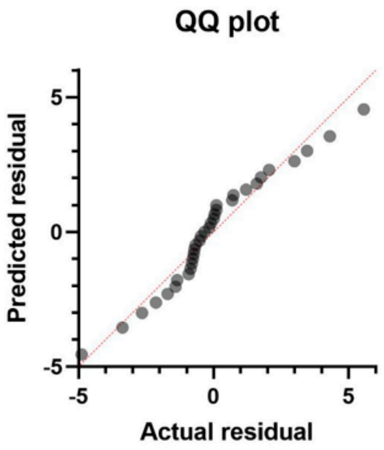

Brown-Forsythe test	
F (DFn, DFd)	1.136 (10, 22)
P value	0.3810
P value summary	ns
Are SDs significantly different (P < 0.05)?	No

AUC of native snake, native lizard, native rodent and reciprocal mutated mimotopes with King Cobra venom from Java.

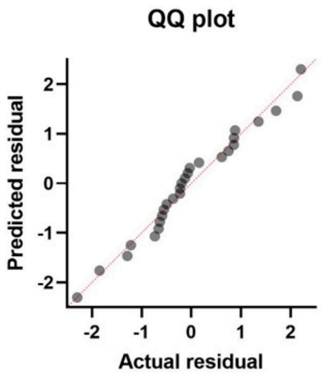

Brown-Forsythe test	
F (DFn, DFd)	0.7035 (8, 18)
P value	0.6850
P value summary	ns
Are SDs significantly different (P < 0.05)?	No

AUC of native snake, native lizard, native rodent and reciprocal mutated mimotopes with King Cobra venom from Cambodia.
